# The LMCE5 unselected cohort of 25 children consecutively diagnosed with untreated stage 4 neuroblastoma over 1 year at diagnosis

**DOI:** 10.1038/sj.bjc.6600627

**Published:** 2002-11-12

**Authors:** D Frappaz, D Perol, J Michon, C Berger, C Coze, J L Bernard, J M Zucker, T Philip

**Affiliations:** Department of Pediatrics, Centre Léon Bérard, 28 rue Laënnec, Lyon, 69373, France; Department of Statistics, Centre Léon Bérard, 28 rue Laënnec, Lyon, 69373, France; Institut Curie, 26 Rue d'Ulm, Paris, France; Hôpital Nord, Av. A. Raimond, Saint Etienne, 42277, France; Hôpital de la Timone, Av. Jean Moulin, Marseille, 13385, France

**Keywords:** autologous bone marrow transplantation, chemotherapy, children, megatherapy, metastasis, neuroblastoma, peripheral blood stem cell, total body irradiation

## Abstract

The Lyon-Marseille-Curie-Est (LMCE) of France cooperative group has previously reported successive series of unselected stage four children older than 1 year at diagnosis with metastatic neuroblastoma (LMCE 1 and 3). The goal of LMCE 5 study was to increase progression free survival rate as compared to LMCE 1 and 3. Based on improvements reported with post induction chemotherapy, the LMCE 5 used post induction for all children, but omitted total body irradiation and immunomagnetic purging in megatherapy regimen for all children. Twenty-five sequentially diagnosed children received an induction regimen which compared with previous induction included an increased dose of etoposide and cyclophosphamide, delivered similar dose of cisplatinum, and deleted doxorubicin and vincristin. After surgery treatment was stratified based on response and eligible children received etoposide carboplatin (LMCE 5A : *n*=10)±doxorubicin (LMCE 5B–C *n*=13) followed by megatherapy (melphalan without total body irradiation and unpurged peripheral blood stem cell rescue). The increase in drug doses during induction did not improve remission rate. The progression free survival at 6 years is 8%. It is significantly worse than LMCE 3, and equivalent to LMCE 1 study though toxic death rate has decreased with increasing experience. Failure to improve the response rate during induction and reducing the megatherapy regimen may be the main factors in this disappointing result. Modified strategies for induction, non toxic alternative to total body irradiation, and post megatherapy regimen should be developed.

*British Journal of Cancer* (2002) **87**, 1197–1203. doi:10.1038/sj.bjc.6600627 www.bjcancer.com

© 2002 Cancer Research UK

## 

Metastatic neuroblastoma (NB) occurring in children older than 1 year remains a challenge in pediatric oncology, although the survival rate of children has progressively increased during the past 20 years (Hartmann *et al*, 1983; Berthold *et al*, 1997; Castleberry *et al*, 1997). Increased doses during induction chemotherapy and/or megatherapy consolidation may both account for this improvement. More recently, the introduction of innovative post consolidation treatment by non cytotoxic drugs (such a retinoic acid) has clarified the role of this biological agent, but may further complicate the interpretation of improved survival curves (Matthay *et al*, 1999). The Lyon-Marseille-Curie East of France group (LMCE) previously published the LMCE1 and LMCE3 studies. These concerned unselected cohorts of children successively seen at diagnosis with metastatic NB (Philip *et al*, 1987, 1991; Frappaz *et al*, 2000). The LMCE1 used a single strategy: all patients received similar induction chemotherapy followed by surgery and a vincristine–melphalan–Total Body Irradiation (TBI) consolidation regimen in chemosensitive patients (Philip *et al*, 1987, 1991). This study and the report of the European Bone Marrow Transplantation Study Group (Ladenstein *et al*, 1993) suggested that clearance of skeletal uptake on bone scan after induction therapy defines a better prognosis subgroup that contains 40% of the population and have a 40% PFS at 5 years (*versus* 10%). The LMCE3 used a higher dose-intensity during induction (doubling of cisplatin dose) (Coze *et al*, 1997) followed by a similar consolidation regimen. For patients who were not in remission of metastases, post induction chemotherapy was delivered prior to consolidation (Frappaz *et al*, 1992; Philip *et al*, 1993). It included either a VP-carboplatinum combination or a first megatherapy. Overall, the 7 years PFS of this cohort was 29%. This was significantly better than the LMCE1 results (8% at 14 years) and than historical controls (10% at 5 years). It suggested that increasing induction and delivering post induction therapy prior to megatherapy was worthwhile. However, when the cure rate increases, quality of life becomes a predominant problem. Total Body Irradiation is responsible for many long term toxicities that may become unacceptable (Neve *et al*, 1999; Peper *et al*, 2000).

The LMCE5 strategy reported here was thus designed to: (1) Further increase total dose and dose intensity of cyclophosphamide and etoposide during induction, with deletion of anthracyclines; (2) Deliver post induction to every patient, and modify the regimen in poor responders; (3) Decrease late sequelae by omitting TBI in the conditioning regimen prior to autologous stem cell transplantation.

## PATIENTS AND METHODS

From December 1992 to April 1995, every patient older than one year with stage 4 NB admitted in the LMCE institutions of Lyon (Centre Léon Bérard), Marseille (Hôpital de la Timone), Curie Institute (Paris), and St. Etienne (Hôpital Nord) entered in the LMCE5 strategy (*n*=25). Staging at diagnosis and during follow-up followed International Neuroblastoma Staging System recommendations (Brodeur *et al*, 1993).

### Patients

Pretreatment investigations included: complete physical examination, full blood count, renal and hepatic function tests, serum LDH NSE and ferritin, and urinary catecholamine determinations. Bone marrow involvement was assessed as previously described using at least two interpretable smears and two interpretable bone marrow biopsies and immunological pool (Favrot *et al*, 1986). MYCN amplification was determined on the primary by open or fine needle biopsy or on the metastatic sites. MIBG scan and measurements of primary and metastatic lesions by ultrasonography and/or CT scan were performed as required. Technecium bone scan was mandatory only in patients with no MIBG uptake.

Response rate and remission status were evaluated after each step of the strategy (induction, post induction and megatherapy). The International Neuroblastoma Response Criteria were used to define response as follows: a complete response (CR) was defined as the disappearance of signs of tumour in both primary and metastatic sites. A continuous complete remission (CCR) was defined as patients who remained in CR. A very good partial remission (VGPR) was defined as a >90% response locally and a complete response elsewhere. A partial response (PR) was defined as more than 50% reduction in both size of the primary tumor and number of metastatic lesions. All regression of tumour <50% were considered as no response (NR). A progressive disease (PD) as more than 25% increase in the size of measurable lesions at any involved site and/or appearance of new lesions. A mixture of CR and/or PR and NR without progression, was defined as mixed response (MR). The response rate was defined as the percentage of CR+VGPR+PR among evaluable patients.

Toxicity was assessed at each step and during the 100 days following consolidation, according to WHO criteria (World Health Organization, 1979).

### Treatment

**Figure 1:**
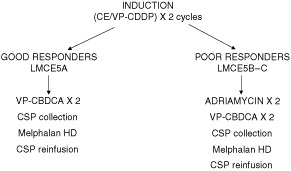
Summary of the protocol.

All patients included in the LMCE5 strategy received induction therapy with the NB92 protocol (Figure 1

).

The induction regimen comprised two cycles alternating CE (Cyclophosphamide (Endoxan® 2000 mg m^−2^ per day, Days 2–4 as a 1 h infusion), Etoposide (Vepeside® 50 mg m^−2^, Days 1–5 as a continuous infusion) with etoposide-cisplatinum (Vepeside® 100 mg m^−2^ as a 1 h infusion and cisplatinum 40 mg m^−2^ days 1–5 as a continuous infusion), delivered at 3 week intervals.

Patients with progressive disease during induction were excluded from further LMCE5 strategy. All other patients underwent surgery after completion of induction. Local radiotherapy was not to be given in this protocol. All underwent steady state collection of autologous peripheral blood stem cell without purging after completion of induction.

The post induction strategy was tailored according to remission status. The patients in CR-VGPR **(**LMCE5A**)** received two courses of etoposide-carboplatin (Vepeside® 100 mg m^−2^ and carboplatin 160 mg m^−2^ Days 1–5) and proceeded to consolidation by high dose melphalan (200 mg m^−2^) with progenitor rescue following surgery.

All other patients underwent a phase II study with two courses of Doxorubicin (Adriamycine® 90 mg m^−2^ in continuous infusion). Non progressive patients then received two courses of etoposide-carboplatin followed by consolidation by high dose melphalan (200 mg m^−2^) with progenitor rescue **(**LMCE5B**)** following surgery. For patients who did not respond to doxorubicin, no further guidelines were proposed but patients could be treated with a similar strategy **(**LMCE5C**)**. Follow-up strategy used routine CT scan, MIBG and catecholamine assessment.

### Statistical analysis

The main aim of this pilot study was to improve survival by giving a combination of post induction strategy adjusted for initial response while decreasing consolidation regimen to improve quality of survival. Objectives were to study Overall Survival (OS), Progression Free Survival (PFS) and toxicity into the three consolidation groups defined prior post-induction, i.e. LMCE5A, 5B and 5C. The intent was to include every child with newly diagnosed metastatic neuroblastoma in a period of 2 years. A stopping rule on toxic death was established. OS and PFS distributions were estimated using the Kaplan–Meier method (Kaplan and Meier, 1958), and compared by the log-rank test (Mantel, 1966). Survival time was defined as the time from the date of diagnosis to the date of death (OS), disease progression or death regardless of cause (PFS) or last follow-up date. All analysis were performed with the SPSS statistical software package (SPSS Inc., Chicago, IL, USA). These data were compared with historical series issued from the same group: LMCE1 (updated) and LMCE3. In the LMCE1, most patients had skeletal assessment with technecium while in LMCE3, all had MIBG.

## RESULTS

From December 1992 to April 1995, 25 patients older than one year were admitted for untreated stage 4 NB in the LMCE participating institutions. No stage 3 patient was included. No patient was excluded.

Sex ratio was seven females/18 males. Median age at diagnosis was 31 (13–167) months. This was not different from previous LMCE 1 and 3 cohort: *P*=0.39. Primary tumour was in retroperitoneum (22), soft tissues of thorax and axillar (1), or absent (2). Its size using INSS criteria was T1 (3), T2 (10), T3 (10), TX (2).

Serum levels of LDH were raised in 24 out of 25 (median 1340 ui; 383–6086), ferritin in 13 of 19 (265 mg ml^−1^; 80–1000) and Neuron Specific Enolase in 21 of 21 (142 mg ml^−1^: 31–622). Urinary levels of catecholamine were raised in 24 of 25, with HVA (median 69 mmol mmol^−1^ of creatinin, 3–350), VMA (25 mmol mmol^−1^, 2–414) and dopamine (4754 mmol mmol^−1^, 86–48 223). Shimada classification was not determined since chemotherapy was delivered prior to surgery. MYCN was amplified in seven out of 24 tested patients (all more than 10 copies). Tumour was diploid in nine out of 12 tested patients.

The MIBG scans used I^123^ in 14 patients and I^131^ in 11 patients. Skeletal uptake was detected by MIBG (21 patients) or technecium bone scan only (one patient). Twenty-four had bone marrow invasion. One patient had neither bone, nor bone marrow involvement but an abdominal mass with thoracic metastases. Other metastatic sites included thorax (one), distant lymph nodes (two).

These data were comparable to that of our previous prospective protocols (LMCE1 and 3) as far as age, sex, LDH levels, MYCN amplification at diagnosis.

### Response

#### Induction therapy

Twenty-five patients received at least one course and 24 patients received the four courses of the induction regimen (one patient with renal toxicity of cisplatinum was excluded from further LMCE5 strategy). The response rate prior to surgery was 19 out of 24 (CR in one, VGPR in one, and PR in 17 patients). Response rate was 20 out of 22 for primary tumours, 23 out of 24 (96 %) for bone marrow invasion and in eight out of 21 (38%) of evaluable skeletal MIBG uptake had completely disappeared. This rate of complete skeletal uptake normalisation did not differ from LMCE1 (35 out of 72: *P*=0.4) and LMCE3: (48 out of 99: *P*=0.39).

#### Surgery

Three patients had undergone frontline surgery of the primary (*n*=2), or for spinal compression (*n*=1). Out of 21 patients who were operated after induction, the tumour was completely removed (*n*=13), or only partially resected (*n*=8). A nephrectomy was required in four out of 20 of patients with abdominal primaries. One patient died of surgical related complication (the operative resection was not evaluable).

The response rate after induction and surgery was 20 out of 23 with five CR, three VGPR, 12 PR (10 had persistent skeletal uptake), two MR-NR and one PD (all had persistent skeletal uptake).

#### Post-induction strategy

Two patients did not fulfil the requirements for inclusion in the post induction strategy. The first one had toxicity of cisplatinum during induction, that was replaced by carboplatin. He then received megatherapy by busulfan–melphalan, and relapsed at 8 months post diagnosis. The other one was a post surgical death.

Twenty three patients were considered to fit with the requirements for post induction strategy. LMCE5A was delivered to 10 patients. Status prior to post induction was: CR (*n*=5), VGPR (*n*=3), PR (*n*=2 by protocol violation). Status post consolidation was : CCR (*n*=5), CR (*n*=2), VGPR (*n*=2), PD (*n*=1). Eight patients eventually progressed and died of disease, two patients are alive disease free. These two patients were 1 and 4 years old at diagnosis respectively. Both underwent initial surgery, and had metastasis in bone marrow and skeleton at diagnosis; one had MYCN amplification.

LMCE5B was delivered to 10 patients who were all in PR. Status post consolidation was: CR (*n*=3), PR (*n*=5) and PD (*n*=1). One patient died of adenoviral infection during Doxorubicin. Eight patients eventually progressed and died of disease, one patient is alive in partial remission.

LMCE5C was used in three patients. Status prior to post induction was NR (*n*=2) or PD (*n*=1). None of these patients responded to the post induction-consolidation strategy and all ultimately progressed and died.

Doxorubicin activity could be evaluated in 12 patients (one patient further died of acute adenoviral infection) and resulted in two complete remissions (16%), one partial remission, six stable disease and three progressive disease. The response rate is thus three out of 12 evaluable patients.

All together, the site of relapse was exclusively primary in only one out of 19 patients: five out of 19 (26%) had local progression, 18 out of 19 (95%) metastatic progression.

### Progression free survival

**Figure 2:**
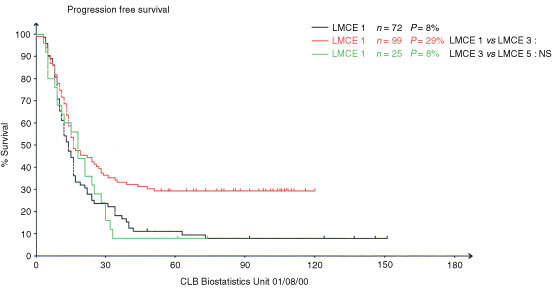
Progression free survival of the three successive LMCE cohorts : LMCE1–3–5.

**Figure 3:**
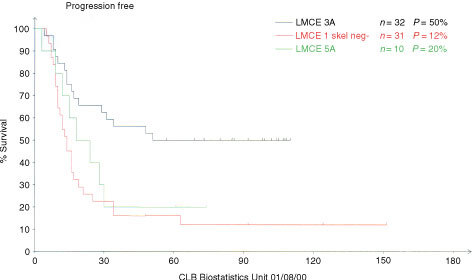
Progression free survival of the favourable subgroup of patients with clearance of skeletal uptake post induction in the three successive LMCE cohorts : LMCE1–3–5.

The Progression free survival (PFS) in the unselected cohort of 25 patients is 8% at 6 years from diagnosis. There was no difference on univariate analysis between patients in CR-VGPR after induction *vs* others, nor those who had cleared their skeletal uptake after induction therapy, nor those more or less than 2 years old at diagnosis. These results compare with a similar cohort of 72 patients previously reported by our group (LMCE1 8% of PFS at 14 years) (Philip *et al*, 1991). However, it is worse than the cohort of 99 patients included in LMCE3 study: 29% at 7 years from diagnosis: *P*<0.07 (Figure 2

) (Frappaz *et al*, 2000). The toxic death rate was decreased with time: 22% (16 out of 72 patients), 10% (10 out of 99 patients) and 4% (one out of 25 patients) in respectively LMCE1, 3 and 5. If only the subgroup of patients who showed normalisation of skeletal uptake after induction are analysed, the progression free is respectively 12, 50 and 20% (Figure 3

) and the toxic death rate 35, 3 and 0% for LMCE1, 3 and 5. There was no difference in PFS between children with or without MYCN amplification.

## DISCUSSION

The LMCE cooperative group organised three successive studies (LMCE1, 3 and 5). The aim of these studies was to enrol every child presenting with metastatic neuroblastoma in each of the participating institutions and in this way to report unbiased series. The LMCE1 study reported a cohort of 72 children, who received standardised induction and megatherapy (Philip *et al*, 1987, 1991). It demonstrated that after induction, the subgroup of children who showed persistent signs of metastases on bone scan had significantly shorter survival. The LMCE3 study was built on such finding (Frappaz *et al*, 2000). It included 99 children. After induction, children with negative MIBG uptake on skeleton immediately proceeded to megatherapy (LMCE3A), while those with persisting skeleton uptake received a post induction cycle prior to megatherapy (LMCE3B-C). The LMCE3 resulted in significantly improved PFS as compared to LMCE1. The improvement was mainly attributable to decrease in toxic death rate and increased PFS in the subgroup of poor prognosis children who had received post induction therapy. Thus, LMCE5 incorporated post induction therapy for all children but removed TBI from conditioning regimen. The results are much worse than in LMCE3 study and the possible reasons for this failure will be discussed.

The populations treated in all three successive studies are comparable as far as age, sex and biological characteristics are concerned (Brodeur *et al*, 1997). The population is equally divided between those more and less than 2 years at diagnosis. Nearly all children had increased LDH, and one-third MYCN amplification. Two-thirds were diploid. Twenty-two children had skeletal involvement.

The induction was modified in the successive LMCE studies. The LMCE3 induction delivered twice as much cisplatinum as in LMCE1 induction that used a combination of all drugs shown to be useful in a metaanalysis (Cheung and Heller, 1991). Both inductions resulted, however, in a similar rate of remissions, especially at skeletal sites. The LMCE5 induction gave similar total dose of cisplatinum though with lower dose intensity. The main objective of this induction was to test a dose effect relationship of etoposide and cyclophosphamide (Meresse *et al*, 1993). The total dose of etoposide and cyclophosphamide was increased 2–4-fold (Table 1

**Table 1:**
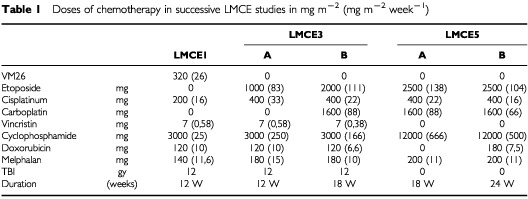
Doses of chemotherapy in successive LMCE studies in mg m^−2^ (mg m^−2^ week^−1^)

). Doxorubicin was deleted only in the favourable group (LMCE5A) and vincristine was omitted for all patients. Here again, the rates of remission were similar, especially on skeleton sites. During the same period, another French cooperative group proposed a similar protocol including doxorubicin, and reached similar results (Plantaz, personal communication). More intensive and prolonged induction strategies have been published, claiming an increased remission rate (Kushner *et al*, 1994). However, increased toxicity was reported. Moreover, these results could not be reproduced by the SFOP group (Valteau-Coinet, personal communication). Thus the dramatic improvements described in high grade lymphoma by increasing the dose of known drugs during induction therapy cannot be translated to metastatic neuroblastoma (Patte *et al*, 1994). It thus seems unlikely that further intensification of induction may result in major improvement of remission rate, as it did not change between three successive cohorts, but this issue is addressed in the recently completed ENSG5 study which compares standard dose of OPEC/OJEC with the rapid COJEC regimen (Pinkerton *et al*, 2000). Either new drugs are to be found, or different scheduling is used, or improvements will come from novel strategies.

Surgical resection was complete in 13 of 21 patients, and one patient died of surgical complications. Much cooperative work has been done in France, between surgeons, radiologists and oncologists, to predict surgical difficulties in localised neuroblastomas (Rubie *et al*, 1998). This has also probably improved surgical management in metastatic disease and explains the low rate of nephrectomy (four out of 20 abdominal procedures). Despite the surgical death reported here, it is unlikely that changes in surgery could be responsible for the poor results. The number of patients with at least a local relapse may reflect the absence of radiation either locally or through a TBI. The role of local radiation has however never been demonstrated by randomised studies in this setting.

Ten patients were included in LMCE5A favourable group. Eight out of these 10 progressed or relapsed, giving a 6 years PFS of only 20%. In the LMCE1 and LMCE3 patients this favourable subgroup received no post induction, but a vincristine, melphalan-TBI conditioning followed by immunobead purged marrow rescue. The PFS of these patients was 12% in LMCE1 and 50% at 7 years in LMCE3. Although numbers are small, the comparison between the successive favourable groups deserves several comments. There was no selection bias in the LMCE5. Eight out of 10 were in CR-VGPR and none had persisting skeletal involvement in LMCE5A whereas in LMCE3A only 27 out of 32 were in CR-VGPR and two had persisting skeletal uptake. Due to better selection in LMCE5A than in LMCE3A patients should have had a better survival. The use of post induction was suggested as improving outcome in LMCE3B patients compared to the LMCE1 and was thus proposed to all LMCE5 patients. A part from Doxorubicine and vincristine omission, the total amount of chemotherapy delivered prior to megatherapy was thus higher in the LMCE5A. The total duration of treatment was thus longer in LMCE5 A (18 weeks) and apart from cisplatinum dose intensity was higher than in LMCE3A (12 weeks). However, LMCE5A patients had poorer survival than the comparable subgroup of LMCE3A patients.

The other patients were included in LMCE5B (*n*=10) or C (*n*=3). Though anthracyclines are quoted as a major drug in meta analysis (Cheung and Heller, 1991), its exact role was challenged (Ninane *et al*, 1981). We thus took the opportunity to test it in resistant patients. After two cycles of doxorubicin, the response rate (three out of 12) suggests that adriamycin is an active agent in neuroblastoma. However due to small number of patients (less than 14), the classical Gehan rule cannot be applied, and we cannot reliably insure that response rate is more than 20%. All of these 13 patients are currently progressing or dead. The influence of Vincristine deletion may also be discussed as it may be a potent inhibitor of neoangiogenesis (Avramis *et al*, 2001).

Probably of importance is the deletion of total body irradiation. This had been decided both in view of the late effects of radiations in young children (Neve *et al*, 1999; Peper *et al*, 2000) and on the circumstantial evidence from the EBMT database that TBI does not, in fact, seem to improve the outcome (Ladenstein *et al*, 1998). We had hypothesised that it might be replaced by introduction of post induction therapy and a small increase in melphalan dose. We suggest here that this is not the case, though several modifications may also have contributed. In view of marked improvements in supportive measures and in view of well-documented synergistic antitumor effects between 2–3 agents, there is no reason today to limit megatherapy to a single agent.

The role of megatherapy has been largely debated. Some non randomised studies show promising results with prolonged intensive standard chemotherapy without massive chemotherapy (Kushner *et al*, 1994). Two randomised studies suggest that megatherapy significantly increases the chance of definitive cure. In a prospectively randomized high risk group of responding patients, a significant advantage for the group treated with high dose Melphalan was demonstrated (Pinkerton, 1991). More recently, the CCG confirmed that high risk neuroblastoma benefited from high dose regimen including TBI and followed by purged autologous marrow reinfusion (Matthay *et al*, 1999). Though there is no clear evidence that TBI containing regimens provide better results than melphalan alone (Ladenstein *et al*, 1998), this is suggested from LMCE5. We had discarded TBI in view of the late sequelae observed in young children: endocrinopathies, growth delays, pulmonary dysfunction, ocular toxicities. The dilemma is thus whether TBI induced sequelae sufficiently severe to justify a clear decrease in survival or can TBI be replaced by something else? The answer may be with busulfan containing regimens (Hartmann *et al*, 1999) or from tandem therapies as used in some LMCE3 patients (Philip *et al*, 1993; Grupp *et al*, 2000). However, high dose busulfan is responsible for acute visceral toxicities, and for delayed endocrinological toxicities (Hartmann *et al*, 1987). Longer follow-up is probably warranted to ensure that no unexpected delayed effect will appear.

A final difference between LMCE1-3 and LMCE5 should be considered. In the latter protocol, immunobead purging (Combaret *et al*, 1989) was omitted. Potentially contaminated progenitor collection remains an unsolved problem. The demonstration that neomycin resistance gene transfected genes in reinfused marrow may be detected in relapsed disease suggests that contaminated marrow may at least contribute to further relapse (Rill *et al*, 1994). Moreover, pulmonary (Glorieux *et al*, 1986) or intracranial (Frappaz *et al*, 1994) relapses after ABMT may suggest that reinfused malignant cells are clonogenic *in vivo*. However, the type of relapses observed in this study were not unusual. The ideal study randomising purged *vs* unpurged marrow, or autologous versus allogenic rescue is lacking. The possible role in the poor results of lack of therapy for minimal residual disease should be stressed. However, the positive results of the randomised CGG study (Matthay *et al*, 1999) was not known at that time. Moreover, none of previous LMCE strategies had incorporated such treatment.

It may thus be concluded that in stage IV neuroblastoma more than 1 year old at diagnosis, the dose effect during induction has been fully explored in the last 20 years without major improvements. The time to explore alternative schedules during induction may have come. The deletion of TBI in a strategy based on consolidation by megatherapy may be detrimental. The addition of post megatherapy treatment such as with novel retinoids and passive or active immunotherapy requires exploration.
